# Comparison of Carotid‐Femoral and Brachial‐Ankle Pulse‐Wave Velocity in Association With Target Organ Damage in the Community‐Dwelling Elderly Chinese: The Northern Shanghai Study

**DOI:** 10.1161/JAHA.116.004168

**Published:** 2017-02-20

**Authors:** Yuyan Lu, Mengyun Zhu, Bin Bai, Chen Chi, Shikai Yu, Jiadela Teliewubai, Henry Xu, Kai Wang, Jing Xiong, Yiwu Zhou, Hongwei Ji, Ximin Fan, Xuejing Yu, Jue Li, Jacques Blacher, Yi Zhang, Yawei Xu

**Affiliations:** ^1^ Department of Cardiology Shanghai Tenth People's Hospital Tongji University School of Medicine Shanghai China; ^2^ Department of Prevention Tongji University School of Medicine Shanghai China; ^3^ Key Laboratory of Arrhythmias, Ministry of Education Tongji University School of Medicine Shanghai China; ^4^ Diagnosis and Therapeutic Center Hôtel‐Dieu AP‐HP Paris Descartes University Paris France

**Keywords:** brachial‐ankle pulse‐wave velocity, cardiovascular disease risk factors, carotid‐femoral pulse‐wave velocity, target organ damage, Epidemiology

## Abstract

**Background:**

Carotid‐femoral pulse‐wave velocity (cf‐PWV) and brachial‐ankle PWV (ba‐PWV) are the 2 most frequently applied PWV measurements. However, little is known about the comparison of hypertensive target organ damage (TOD) with cf‐PWV and ba‐PWV.

**Methods and Results:**

A total of 1599 community‐dwelling elderly subjects (age >65 years) in northern Shanghai were recruited from June 2014 to August 2015. Both cf‐PWV and ba‐PWV were measured using SphygmoCor and VP1000 systems, respectively. Within the framework of comprehensive cardiovascular examinations, risk factors were assessed, and asymptomatic TOD, including left ventricular mass index, peak transmitral pulsed Doppler velocity/early diastolic tissue Doppler velocity (E/Ea), carotid intima‐media thickness, arterial plaque, creatinine clearance rate, and urinary albumin‐creatinine ratio were all evaluated. Both PWVs were significantly associated with male sex, age, waist/hip circumference, fasting plasma glucose, and systolic blood pressure, and ba‐PWV was also significantly related to body mass index. Both PWVs were significantly correlated with most TOD. When cf‐PWV and ba‐PWV were both or separately put into the stepwise linear regression model together with cardiovascular risk factors and treatment, only cf‐PWV, but not ba‐PWV, was significantly associated with carotid intima‐media thickness and creatinine clearance rate (*P*<0.05). When cf‐PWV and ba‐PWV were both or separately put into the same full‐mode model after adjustment for confounders, only cf‐PWV, but not ba‐PWV, showed significant association with carotid intima‐media thickness and creatinine clearance rate (*P*<0.05). Similar results were observed in logistic regression analysis.

**Conclusions:**

Taken together, in the community‐dwelling elderly Chinese, cf‐PWV seems to be more closely associated with hypertensive TOD, especially vascular and renal TOD, as compared with ba‐PWV.

**Clinical Trial Registration:**

URL: http://www.clinicaltrials.gov. Unique identifier: NCT02368938.

## Introduction

Pulse‐wave velocity (PWV) has been widely accepted in clinical practice as a gold standard of arterial stiffness,^1^ which contributes to vascular diseases through phasic mechanical stresses and stretch imposed on vessels as well as shear stress and endothelial dysfunction.^2^, ^3^ Carotid‐femoral PWV (cf‐PWV) and brachial‐ankle PWV (ba‐PWV) are the 2 most frequently applied PWV measurements. The former is recommended as a clinical marker for cardiovascular risk stratification in hypertensives by the European Society of Hypertension (ESH) and European Society of Cardiology (ESC)^4^, ^5^ as well as the American Heart Association,^6^ whereas the latter is widely used and investigated in Asia.^2^, ^7^, ^8^

As a velocity, PWV can be conveniently measured as the distance divided by the time interval between 2 selected points of the arterial system.^5^ Of note, unlike cf‐PWV, there is no real artery passing through the 2 selected points in the calculation of ba‐PWV. Because ba‐PWV is an estimated velocity, and the “path” contains both elastic and muscular arteries,^5^ cf‐PWV traveling through only the elastic aorta should be regarded as a better indicator. The cf‐PWV has been validated for its prognostic significance of future cardiovascular events in various clinical investigations, such as patients with hypertension, diabetes, and renal failure, the general population, and apparently healthy subjects.^4^, ^9^, ^10^, ^11^, ^12^, ^13^, ^14^, ^15^ Nevertheless, Sheng et al^16^ indicated that, in 3876 participants of whom 2292 (59.1%) were hypertensive patients, ba‐PWV predicted mortality in elderly Chinese who showed markedly increased PWV and hypertension during the median follow‐up of 5.9 years. Consistently, Yu et al^2^ also observed that, in 86 apparently healthy subjects and 234 patients with various degrees of abnormality in cardiac structure and function, ba‐PWV was more representative of arterial load of the left ventricle (LV) than cf‐PWV, probably because ba‐PWV encompasses a greater extent of the arterial tree than cf‐PWV. Thus, it is controversial whether cf‐PWV or ba‐PWV is more representative of arterial stiffness and which PWV has a more pronounced prognostic value. Because asymptomatic hypertensive target organ damage (TOD) is a very prevalent and critical prodrome of cardiovascular events and mortality as well as arterial aging, we investigated the association of hypertensive TOD with cf‐PWV and ba‐PWV within the framework of cardiovascular risk assessment in a community‐based elderly cohort.

## Methods

### Study Design

The Northern Shanghai Study is a prospective, ongoing, and multistage cohort study with the objective of investigating the cardiovascular risk assessment system in elderly Chinese. From June 2014 to August 2015, subjects were recruited if they were (1) aged 65 years or more, (2) local residents from urban communities in the north of Shanghai, and (3) available for long‐term follow‐up. Exclusion criteria included (1) severe cardiac disease (NewYork Heart Association Class IV) or end‐stage renal disease (chronic kidney disease >4), (2) malignant tumor with life expectancy less than 5 years, and (3) stroke history within 3 months. Finally, 1721 subjects were invited, of whom 1599 (92.9%) were enrolled. The Northern Shanghai Study was authorized and financially supported by the Shanghai municipal government (Grant ID 2013ZYJB0902) and was approved by the Ethics Committee of Shanghai Tenth People's Hospital. Written informed consent was obtained from all subjects after relevant information had been provided to them and their relatives.

### Social, Clinical, and Biological Parameters

As previously described,^17^ information obtained from the questionnaire contained sex, age, weight, and height, smoking habits, family history of premature cardiovascular diseases (CVD), history of diabetes mellitus, hypertension, cardio‐ or cerebrovascular diseases, renal diseases, and usage of medications.

Venous blood samples and urine samples were obtained in subjects after an overnight fast. Biological markers, including plasma low‐density lipoprotein (LDL) cholesterol, high‐density lipoprotein (HDL) cholesterol, plasma creatinine, and urinary microalbumin and creatinine were assayed by standard methods in the Department of Laboratory Medicine of Shanghai Tenth People's Hospital. Fasting plasma glucose was measured with the glucose‐oxidase method. Creatinine clearance rate (CCR) was calculated by the modified Modification of Diet in Renal Disease formula for Chinese as follows: CCR (mL/[min·1.73 m^2^])=175×(plasma creatinine)^−1.234^×age^−0.179^ (women×0.79).^18^ Urinary microalbumin divided by urinary creatinine was defined as urinary albumin‐creatinine ratio (UACR).

### Measurement of Blood Pressures and Ankle‐Brachial Index

Blood pressure (BP) of each subject was measured in the morning with the electronic device by specialized physicians 3 times after at least 10 minutes of rest in the sitting position. The average of the 3 BP readings was calculated and used in the subsequent statistical analysis. Pulse pressure was defined as the difference between systolic (SBP) and diastolic BP.

Bilateral brachial and ankle BPs were automatically and simultaneously measured to calculate the ankle‐brachial index (calculated as ankle SBP divided by brachial SBP) using the VP1000 system (Omron, Kyoto, Japan).

### Pulse‐Wave Velocity

The order of cf‐PWV and ba‐PWV measurements was randomly chosen by the coin‐tossing method. The cf‐PWV was measured using applanation tonometry (SphygmoCor, AtCor Medical, Sydney, Australia), according to the European Expert Consensus on Arterial Stiffness.^19^ First, peripheral BP was recorded twice using the Omron device with an interval of 3 minutes after a rest about 10 minutes. Second, the superficial distance covered by the pulse‐wave was measured directly from the carotid to the femoral artery. Third, pressure waveforms in the right carotid and right femoral arteries were recorded for each subject, and transit time for each artery was automatically calculated via ECG data by the “foot‐to‐foot” method. Finally, cf‐PWV was calculated by traveling distance divided by traveling time, and an operator index greater than 80% indicated high‐quality waveforms.

The ba‐PWV was measured using the VP1000 system (Omron) as previously described.^16^ In brief, pulse waves of brachial and posterior tibial arteries were measured via pressure cuffs on both arms and both ankles in the supine position after a 10‐minute rest. The device estimated the travel path from body height and calculated ba‐PWV automatically as the travel path divided by the time difference between brachial and ankle arterial pulse waves. Right ba‐PWV was used for analysis in the present study. Of note, ba‐PWV was excluded when ankle‐brachial index was <0.9, which is considered indicative of peripheral arterial diseases.

### Ultrasonography

The common carotid artery intima‐media thickness (CIMT) was measured using the MyLab 30 Gold cardiovascular system (ESAOTE SpA, Genoa, Italy) with a 7.5‐MHz probe. As previously described,^1^ measurements were taken on the left common carotid artery 2 cm from the bifurcation and were always performed on plaque‐free arterial segments. Three measurements of CIMT were taken, and the average value was used for further analysis. Simultaneously, the presence or absence of plaques in the left and right carotid arteries was recorded.

M‐mode and 2‐dimensional echocardiography were performed using the same device with a 3.5‐MHz probe, according to the guidelines of the American Society of Echocardiography.^20^ LV ejection fraction was measured by M‐mode echocardiography using the adjusted Teicholz formula.^21^ Left ventricular end‐diastolic diameter (LVEDd), interventricular septal (IVSd), and posterior wall thickness at end‐diastole (PWTd) were measured by M‐mode or 2‐dimensional echocardiography from the parasternal view^17^, ^20^ and then used to calculate left ventricular mass (LVM),^22^ which was further standardized for body size as left ventricular mass index (LVMI) by dividing by body surface area (BSA).

$$\text{LVM}\;\left(g\right)=0.8\times \{1.04\times \left[{\left(\text{LVEDd}+\text{PWTd}+\text{IVSd}\right)}^{3}-{\left(\text{LVEDd}\right)}^{3}\right]\}+0.6$$

and

$$\text{LVMI}\;(g/m^{2})=\text{LVM}÷\text{BSA}$$

Transmitral flow velocity was detected by 2‐dimensional Doppler echocardiography according to the American Society of Echocardiography^23^: peak transmitral pulsed Doppler velocity/early diastolic tissue Doppler velocity (E/Ea) was calculated for the evaluation of LV diastolic function. In addition, left atrial volume was calculated using the model formula and standardized to body size by dividing by BSA to give the left atrial volume index (LAVI):

$$\text{LAVI}\;\left(\text{mL}/m^{2}\right)=\left[\pi \times \left(\text{SA1}\times \text{SA2}\times \text{LA}\right)/6\right]÷\text{BSA}$$

where SA1 is the M‐mode left atrial dimension in the parasternal short‐axis view, and SA2 and LA are measurements of short and long axes in the apical 4‐chamber view at ventricular end‐systole.^24^ All ultrasonographic measurements were performed by a single experienced sonographer.

### Definition of Hypertensive TOD

Asymptomatic hypertensive TOD include cardiac, arterial, and renal TOD. Left ventricular hypertrophy was defined as LVMI ≥115 g/m^2^ (male) or LVMI ≥95 g/m^2^ (female). LV diastolic dysfunction was assessed by E/Ea and other evidence of abnormal LV relaxing and filling, such as enlarged left atrial volume and increased LVM.^24^ Specifically, diastolic dysfunction was defined as E/Ea≥15, or 8<E/Ea<15 with any of the following: LAVI>40 mL/m^2^ or LVMI>149 g/m^2^ (male) or LVMI≥122 g/m^2^ (female).^24^, ^25^ Arterial TOD was defined as increased CIMT (CIMT>900 μm) or as the presence of arterial plaque,^26^ chronic kidney diseases (CCR<60 mL/[min·1.73 m^2^), and microalbuminuria (UACR >30).

### Statistical Analysis

Quantitative and qualitative parameters were presented as means±standard deviation and numbers with the percentage in parentheses, compared between men and women by Student t test and chi‐squared test, respectively. Multivariate linear regressions were conducted to detect the association of conventional cardiovascular risk factors with cf‐PWV and ba‐PWV, such as sex, age, smoking, family history of premature CVD, waist/hip circumference, body mass index (BMI), fasting plasma glucose, LDL cholesterol, HDL cholesterol, and SBP. Pearson correlation analysis was applied to investigate the correlation of asymptomatic hypertensive TOD with cf‐PWV and ba‐PWV, respectively. Further, stepwise multivariate linear and logistic regressions were conducted to investigate the association of hypertensive TOD with cf‐PWV and ba‐PWV. Only variables staying in the final model were presented. In addition, cf‐PWV and ba‐PWV were both or separately put into the same full‐mode regression models after adjustments for confounders to detect the regression coefficients and odds ratios (ORs) of PWVs in hypertensive TOD. It is noteworthy that age and SBP were forced into all models of regressions because PWVs were strongly dependent on the 2 variables. Statistical analysis was performed using SAS software, version 9.3 (SAS Institute, Cary, NC). *P*<0.05 was considered statistically significant.

## Results

### Participants

Characteristics of participants by sex are presented in Table 1, including conventional cardiovascular risk factors, asymptomatic hypertensive TOD, and diseases and treatment. The 1599 participants included 711 (44.5%) men, 312 (19.5%) participants with diabetes, 843 (52.7%) participants with hypertension, of whom 799 (93.9%) were taking antihypertensive agents. Men, compared with women, had significantly more smokers (49.4% vs 1.7%, *P*<0.001), higher waist circumference (87.8±9.8 vs 84.1±9.4 cm, *P*<0.001), lower incidence of family history of premature CVD (17.4% vs 22.6%, *P*=0.01), lower HDL cholesterol (1.28±0.33 vs 1.46±0.36 mmol/L, *P*<0.001), lower LDL cholesterol (3.04±0.85 vs 3.33±0.83 mmol/L, *P*<0.001), lower pulse pressure (54.4±14.0 vs 56.2±16.1 mm Hg, *P*=0.02), higher CIMT (633.0±159.5 vs 595.5±136.4 μm, *P*<0.001), lower CCR (88.3±20.0 vs 95.8±22.5 mL/[min·1.73 m^2^], *P*<0.001), and lower ba‐PWV (1838.1±355.9 vs 1895.0±393.9 cm/s, *P*=0.01).

**Table 1:** Characteristics of Participants by Sex

	Overall (n=1599)	Men(n=711)	Women (n=888)	*P* Value
Cardiovascular risk factors				
Age, y	72.6±6.0	72.7±5.9	72.6±6.1	0.96
Smoker, n (%)	366 (22.9)	351 (49.4)	15 (1.7)	<0.001^a^
Family history of premature CVD, n (%)	324 (20.3)	124 (17.4)	200 (22.6)	0.01^a^
Waist circumference, cm	85.8±9.7	87.8±9.8	84.1±9.4	<0.001^a^
Hip circumference, cm	97.1±7.2	97.2±6.8	97.0±7.5	0.50
Body mass index, kg/m^2^	23.9±3.5	23.9±3.3	23.9±3.6	0.92
Fasting plasma glucose, mmol/L	5.69±1.70	5.72±1.67	5.67±1.73	0.55
High‐density lipoprotein cholesterol, mmol/L	1.38±0.36	1.28±0.33	1.46±0.36	<0.001^a^
Low‐density lipoprotein cholesterol, mmol/L	3.20±0.85	3.04±0.85	3.33±0.83	<0.001^a^
Systolic blood pressure, mm Hg	134.3±17.7	134.3±16.8	134.3±18.4	0.95
Asymptomatic hypertensive target organ damage				
Pulse pressure, mm Hg	55.4±15.2	54.4±14.0	56.2±16.1	0.02^a^
Left ventricular mass index, g/m^2^	90.0±28.6	90.5±28.8	89.6±28.4	0.54
Carotid intima‐medium thickness, μm	612.1±148.2	633.0±159.5	595.5±136.4	<0.001^a^
Right ankle‐brachial index	1.05±0.13	1.05±0.14	1.05±0.12	0.40
Creatinine clearance rate, mL/[min·1.73 m^2^]	92.4±21.7	88.3±20.0	95.8±22.5	<0.001^a^
Urinary albumin‐creatinine ratio, mg/g	54.9±181.6	51.1±108.3	58.0±224.4	0.45
cf‐PWV, m/s	9.4±2.3	9.3±2.4	9.5±2.2	0.32
ba‐PWV, cm/s	1870.5±379.0	1838.1±355.9	1895.0±393.9	0.01^a^
Diseases and treatment				
Hypertension, n (%)	843 (52.7)	385 (54.2)	458 (51.6)	0.31
Diabetes mellitus, n (%)	312 (19.5)	137 (19.3)	175 (19.7)	0.99
Hyperlipidemia, n (%)	549 (34.3)	231 (32.5)	318 (35.8)	0.16
Antihypertensive treatment, n (%)	801 (50.1)	365 (51.3)	436 (49.1)	0.37
Antidiabetic treatment, n (%)	279 (17.4)	123 (17.3)	156 (17.6)	0.89
Antihyperlipidaemic treatment, n (%)	259 (16.2)	102 (14.3)	157 (17.7)	0.07

Data are means±standard deviation or numbers with percentages in parentheses. Student t test and chi‐squared test were conducted to compare the differences between men and women for quantitative and qualitative variables, respectively. Creatinine clearance rate was calculated with modified Modification of Diet in Renal Disease formula for Chinese. ba‐PWV indicates brachial‐ankle pulse‐wave velocity; cf‐PWV, carotid‐femoral pulse‐wave velocity; CVD, cardiovascular diseases.

a *P*<0.05.

### Association of Cardiovascular Risk Factors With cf‐PWV and ba‐PWV

Conventional cardiovascular risk factors, including sex, age, smoking, family history of premature CVD, waist/hip circumference, BMI, fasting plasma glucose, HDL cholesterol, LDL cholesterol, and SBP, were put into a multivariate linear regression model to investigate their association with cf‐PWV and ba‐PWV. As shown in Table 2, both cf‐PWV and ba‐PWV were significantly and consistently associated with male sex, age, waist/hip circumference, fasting plasma glucose, and SBP (*P*<0.04), whereas ba‐PWV was also significantly related to BMI (*P*=0.007). The total R^2^ values of the full models with cf‐PWV and ba‐PWV were 0.252 and 0.301, respectively.

**Table 2:** Association of Cardiovascular Risk Factors With cf‐PWV and ba‐PWV

Cardiovascular Risk Factors	cf‐PWV			ba‐PWV		
	Regression Coefficient	*P* Value	Incremental R^2^	Regression Coefficient	*P* Value	Incremental R^2^
Sex (1=male, 0=female)	−0.36	0.007^a^	0.003	−83.5	<0.001^a^	0.010
Age, y	0.12	<0.001^a^	0.082	20.4	<0.001^a^	0.091
Smoker (1=smoker, 0=nonsmoker)	0.16	0.29		−15.9	0.55	
Family history of premature CVD (1=yes, 0=no)	0.10	0.45		−9.1	0.68	
Waist/hip circumference	2.5	0.011^a^	0.004	356.0	0.035^a^	0.003
Body mass index, kg/m^2^	0.002	0.92		−8.3	0.007^a^	0.002
Fasting plasma glucose, mmol/L	0.201	<0.001^a^	0.026	26.9	<0.001^a^	0.016
Low‐density lipoprotein cholesterol, mmol/L	0.089	0.15		11.5	0.29	
High‐density lipoprotein cholesterol, mmol/L	−0.26	0.10		−26.2	0.34	
Systolic blood pressure, mm Hg	0.038	<0.001^a^	0.133	8.3	<0.001^a^	0.178
Total *R* ^2^	0.252			0.301		

Multivariate linear regressions were conducted to investigate the association of cardiovascular risk factors with cf‐PWV and ba‐PWV. ba‐PWV indicates brachial‐ankle pulse‐wave velocity; cf‐PWV, carotid‐femoral pulse‐wave velocity; CVD, cardiovascular diseases.

a *P*<0.05.

### Correlation of Asymptomatic Hypertensive TOD With cf‐PWV and ba‐PWV

In the correlation analysis of PWVs with hypertensive TOD, cf‐PWV was significantly correlated with all parameters of asymptomatic TOD (*P*<0.01), and ba‐PWV was also significantly correlated with all parameters (*P*<0.05), except CCR (*P*=0.077) (Table 3).

**Table 3:** Correlation of Asymptomatic Hypertensive Target Organ Damage With cf‐PWV and ba‐PWV

Asymptomatic Hypertensive Target Organ Damage	cf‐PWV		ba‐PWV	
	r	*P* Value	r	*P* Value
Left ventricular mass index	0.12	<0.001^a^	0.11	<0.001^a^
E/Ea	0.07	0.004^a^	0.09	0.001^a^
Carotid intima‐media thickness	0.13	<0.001^a^	0.08	0.003^a^
Creatinine clearance rate	−0.15	<0.001^a^	−0.05	0.077
Urinary albumin‐creatinine ratio	0.09	<0.001^a^	0.06	0.041^a^

Pearson correlation analyses were conducted to investigate the association of asymptomatic hypertensive target organ damage with cf‐PWV and ba‐PWV. ba‐PWV indicates brachial‐ankle pulse‐wave velocity; cf‐PWV, carotid‐femoral pulse‐wave velocity; E/Ea, peak transmitral pulsed Doppler velocity/early diastolic tissue Doppler velocity.

a *P*<0.05.

### Association of cf‐PWV and ba‐PWV With Asymptomatic Hypertensive TOD

Because PWVs were greatly dependent on age and blood pressure, the 2 variables (age and SBP) were forced into all models in the following linear and logistic regression analysis to investigate the association of cf‐PWV and ba‐PWV with hypertensive TOD. As shown in Table 4, when both cf‐PWV and ba‐PWV were put into the stepwise linear regression model, together with cardiovascular risk factors and number of subjects on medications, only cf‐PWV, but not ba‐PWV, was significantly associated with CIMT (5.05±2.03 μm, *P*=0.001, incremental R^2^=0.005) and CCR (−0.60±0.29 mL/[min·1.73 m^2^], *P*=0.041, incremental R^2^=0.003). Consistently, when they were separately put into the same model, only cf‐PWV showed significant association with CIMT (5.11±1.82 μm, *P*=0.005), and CCR (−0.79±0.26 mL/[min·1.73 m^2^], *P*=0.002) (Table 5). In addition, the same results were observed in full‐mode linear regression models after adjustments for confounders, regardless of whether cf‐PWV and ba‐PWV were both or separately put into the models (Figure, panels A and B).

**Table 4:** Determinants of Hypertensive TOD Analyzed by Multivariate Linear Regressions When cf‐PWV and ba‐PWV Are Both Put Into the Same Models

Cardiovascular Risk Factors, Treatment, and PWVs	Cardiac TOD				Vascular TOD		Renal TOD			
	LVMI		E/Ea		CIMT		CCR		UACR	
	β±SE	*P* Value	β±SE	*P* Value	β±SE	*P* Value	β±SE	*P* Value	β±SE	*P* Value
Age, y	0.63±0.13	<0.001	0.01±0.02	0.49	2.55±0.76	<0.001	−1.1±0.1	<0.001	1.8±1.0	0.068
Systolic blood pressure, mm Hg	0.22±0.05	<0.001	0.04±0.01	<0.001	0.39±0.25	0.12	0.14±0.04	<0.001	−0.24±0.35	0.48
Sex (1=male, 0=female)	···	···	−1.01±0.25	<0.001	26.0±10.0	0.01	−7.3±1.2	<0.001	···	···
Smoker (1=smoker, 0=nonsmoker)	···	···	0.67±0.30	0.03	17.7±12.0	0.14	···	···	···	···
Family history of premature CVD (1=yes, 0=no)	···	···	−0.49±0.25	0.053	···	···	···	···	···	···
Waist/hip circumference	39.5±13.5	0.004	···	···	···	···	···	···	···	···
Body mass index, kg/m^2^	0.89±0.26	<0.001	···	···	···	···	···	···	4.0±1.7	0.02
Fasting plasma glucose, mmol/L	−0.92±0.45	0.040	···	···	···	···	1.0±0.4	0.01	5.6±3.2	0.0828
Low‐density lipoprotein cholesterol, mmol/L	···	···	···	···	10.0±4.8	0.04	−1.8±0.7	0.008	···	···
High‐density lipoprotein cholesterol, mmol/L	···	···	···	···	···	···	6.3±1.6	<0.001	···	···
Antihypertensive treatment (1=yes, 0=no)	5.8±1.7	<0.001	0.38±0.22	0.079	···	···	−3.0±1.2	0.017	34.3±11.9	0.004
Antidiabetic treatment (1=yes, 0=no)	···	···	···	···	···	···	3.3±1.7	0.0572	···	···
Antihyperlipidemic treatment (1=yes, 0=no)	···	···	0.54±0.27	0.045	···	···	···	···	···	···
cf‐PWV, m/s	···	···	···	···	5.05±2.03	0.01	−0.60±0.29	0.041	···	···
ba‐PWV, cm/s	···	···	···	···	···	···	···	···	···	···
Total R^2^	0.108		0.061		0.043		0.155		0.020	

Stepwise multivariate linear regressions were conducted to investigate the determinants of hypertensive TOD. Age and systolic blood pressure were forced into all models. Only variables staying in the final model are presented. ··· indicates nonsignificance; ba‐PWV indicates brachial‐ankle pulse‐wave velocity; CCR, creatinine clearance rate; cf‐PWV, carotid‐femoral pulse‐wave velocity; CIMT, carotid intima‐media thickness; CVD, cardiovascular diseases; E/Ea, peak transmitral pulsed Doppler velocity/early diastolic tissue Doppler velocity; LVMI, left ventricular mass index; TOD, target organ damage; UACR, urinary albumin‐creatinine ratio.

**Table 5:** Determinants of Hypertensive TOD Analyzed by Multivariate Linear Regressions When cf‐PWV and ba‐PWV Are Separately Put Into the Same Models

PWVs	Cardiac TOD				Vascular TOD		Renal TOD			
	LVMI		E/Ea		CIMT		CCR		UACR	
	β±SE	*P* Value	β±SE	*P* Value	β±SE	*P* Value	β±SE	*P* Value	β±SE	*P* Value
cf‐PWV, m/s	···	···	···	···	5.11±1.82	0.005	−0.79±0.26	0.002	···	···
ba‐PWV, cm/s	···	···	···	···	···	···	···	···	···	···

Stepwise multivariate linear regressions were conducted to investigate the determinants of hypertensive TOD. Age and systolic blood pressure were forced into all models. Only variables staying in the final model are presented. ··· indicates nonsignificance; ba‐PWV indicates brachial‐ankle pulse‐wave velocity; CCR, creatinine clearance rate; cf‐PWV, carotid‐femoral pulse‐wave velocity; CIMT, carotid intima‐media thickness; E/Ea, peak transmitral pulsed Doppler velocity/early diastolic tissue Doppler velocity; LVMI, left ventricular mass index; TOD, target organ damage; UACR, urinary albumin‐creatinine ratio.

**Figure 1:**
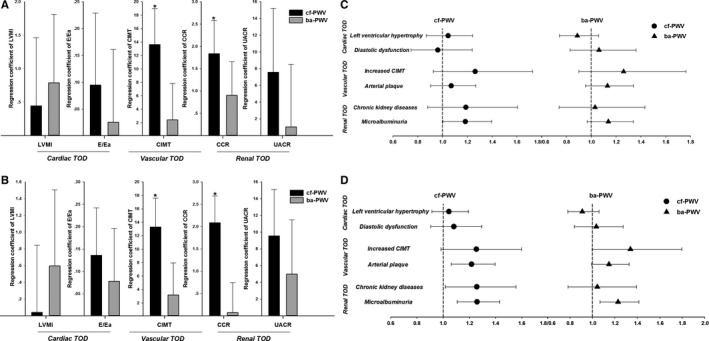
The cf‐PWV and ba‐PWV in association with hypertensive TOD after adjustments for confounders. A and B, Regression coefficients of cf‐PWV and ba‐PWV are presented after adjustments for confounders using multivariate linear regressions when cf‐PWV and ba‐PWV were both (A) or separately (B) put into the same full‐mode model. C and D, Odds ratios of cf‐PWV and ba‐PWV were presented after adjustments for confounders using logistic regressions when cf‐PWV and ba‐PWV were both (C) or separately (D) put into the same full‐mode model. ba‐PWV indicates brachial‐ankle pulse‐wave velocity; CCR, creatinine clearance rate; cf‐PWV, carotid‐femoral pulse‐wave velocity; CIMT, carotid intima‐media thickness; CVD, cardiovascular diseases; E/Ea, peak transmitral pulsed Doppler velocity/early diastolic tissue Doppler velocity; LVMI, left ventricular mass index; TOD, target organ damage; UACR, urinary albumin‐creatinine ratio. Definitions of hypertensive TOD are presented in Methods.

Moreover, when both cf‐PWV and ba‐PWV were put into the stepwise logistic regression model in Table 6, together with conventional cardiovascular risk factors and number of subjects on medications, only cf‐PWV, but not ba‐PWV, was significantly associated with increased CIMT (OR=1.34, 95% CI 1.02‐1.76, *P*=0.037) and microalbuminuria (OR=1.22, 95% CI 1.05‐1.41, *P*=0.009). Similarly, when they were separately put into the stepwise linear regression model, cf‐PWV showed significant association with arterial plaque (OR=1.19, 95% CI 1.03‐1.36, *P*=0.015) and microalbuminuria (OR=1.21, 95% CI 1.06‐1.38, *P*=0.004), whereas ba‐PWV was significantly associated with increased CIMT (OR=1.36, 95% CI 1.01‐1.82, *P*=0.042) and microalbuminuria (OR=1.20, 95% CI 1.04‐1.38, *P*=0.013) (Table 7). Finally, both PWVs were put into the full‐mode logistic regression model after adjustments for confounders, and only cf‐PWV showed a significant correlation with microalbuminuria (OR=1.23, 95% CI 1.07‐1.42, *P*=0.045) (Figure, panel C). However, when they were separately put into the full‐mode logistic regression model, cf‐PWV was significantly related with arterial plaque (OR=1.22, 95% CI 1.06‐1.40, *P*=0.005), chronic kidney disease (OR=1.26, 95% CI 1.02‐1.56, *P*=0.035), and microalbuminuria (OR=1.26, 95% CI 1.11‐1.43, *P*<0.001), whereas ba‐PWV was significantly associated only with microalbuminuria (OR=1.23, 95% CI 1.07‐1.42, *P*=0.004) in Figure, panel D.

**Table 6:** Determinants of Hypertensive TOD Analyzed by Logistic Regressions When cf‐PWV and ba‐PWV Are Both Put Into the Same Models

Cardiovascular Risk Factors, Treatment, and PWVs	Cardiac TOD				Vascular TOD				Renal TOD			
	Left Ventricular Hypertrophy		Diastolic Dysfunction		Increased CIMT		Arterial Plaque		Chronic Kidney Diseases		Microalbuminuria	
	OR (95% CI)	*P* Value	OR (95% CI)	*P* Value	OR (95% CI)	*P* Value	OR (95% CI)	*P* Value	OR (95% CI)	*P* Value	OR (95% CI)	*P* Value
Age (y), +1 SD	1.19 (1.04‐1.37)	0.012	0.99 (0.82‐1.22)	0.987	1.13 (0.84‐1.53)	0.418	1.43 (1.24‐1.63)	<0.001	2.24 (1.74‐2.90)	<0.001	1.13 (0.99‐1.29)	0.075
Systolic blood pressure (mm Hg), +1 SD	1.30 (1.13‐1.50)	<0.001	1.31 (1.07‐1.60)	0.009	0.95 (0.70‐1.29)	0.757	1.07 (0.95‐1.21)	0.276	0.78 (0.57‐1.05)	0.099	1.13 (0.99‐1.30)	0.079
Sex (1=male, 0=female)	0.26 (0.19‐0.35)	<0.001	0.57 (0.38‐0.86)	0.007	···	···	···	···	···	···	0.61 (0.47‐0.79)	<0.001
Smoker (1=smoker, 0=nonsmoker)	···	···	···	···	2.56 (1.41‐4.64)	0.002	1.50 (1.11‐2.03)	0.009	···	···	···	···
Family history of premature CVD (1=yes, 0=no)	···	···	···	···	···	···	···	···	···	···	0.72 (0.52‐0.98)	0.037
Waist/hip circumference, +1 SD	1.29 (1.12‐1.49)	<0.001	···	···	···	···	···	···	···	···	1.23 (1.08‐1.40)	0.003
Body mass index (kg/m^2^), +1 SD	···	···	···	···	···	···	···	···	···	···	···	···
Fasting plasma glucose (mmol/L), +1 SD	···	···	···	···	···	···	···	···	···	···	1.22 (1.07‐1.39)	0.003
Low‐density lipoprotein cholesterol (mmol/L), +1 SD	···	···	···	···	1.31 (1.01‐1.69)	0.042	···	···	···	···	···	···
High‐density lipoprotein cholesterol (mmol/L), +1 SD	···	···	···	···	···	···	···	···	0.72 (0.53‐0.96)	0.028	···	···
Antihypertensive treatment (1=yes, 0=no)	1.56 (1.17‐2.09)	0.003	1.72 (1.12‐2.65)	0.014	···	···	···	···	1.93 (1.05‐3.54)	0.035	1.70 (1.30‐2.22)	<0.001
Antidiabetic treatment (1=yes, 0=no)	···	···	···	···	···	···	1.58 (1.13‐2.21)	0.008	···	···	···	···
Antihyperlipidaemic treatment (1=yes, 0=no)	···	···	···	···	···	···	···	···	···	···	1.63 (1.19‐2.24)	0.003
cf‐PWV (m/s), +1 SD	···	···	···	···	1.34 (1.02‐1.76)	0.037	···	···	···	···	1.22 (1.05‐1.41)	0.009
ba‐PWV (cm/s), +1 SD	···	···	···	···	···	···	···	···	···	···	···	···

Stepwise logistic regressions were conducted to investigate the determinants of hypertensive TOD. Age and systolic blood pressure were forced into all models. Only variables staying in the final model are presented. ··· indicates nonsignificance; ba‐PWV indicates brachial‐ankle pulse‐wave velocity; cf‐PWV, carotid‐femoral pulse‐wave velocity; CIMT, carotid intima‐media thickness; CVD, cardiovascular diseases; OR, odds ratio; TOD, target organ damage. Definitions of hypertensive TOD were presented in Methods.

**Table 7:** Determinants of Hypertensive TOD Analyzed by Logistic Regressions When cf‐PWV and ba‐PWV Are Separately Put Into the Same Models

PWVs	Cardiac TOD				Vascular TOD				Renal TOD			
	Left Ventricular Hypertrophy		Diastolic Dysfunction		Increased CIMT		Arterial Plaque		Chronic Kidney Diseases		Microalbuminuria	
	OR (95% CI)	*P* Value	OR (95% CI)	*P* Value	OR (95% CI)	*P* Value	OR (95% CI)	*P* Value	OR (95% CI)	*P* Value	OR (95% CI)	*P* Value
cf‐PWV (m/s), +1 SD	···	···	···	···	···	···	1.19 (1.03‐1.36)	0.015	···	···	1.21 (1.06‐1.38)	0.004
ba‐PWV (cm/s), +1 SD	···	···	···	···	1.36 (1.01‐1.82)	0.042	···	···	···	···	1.20 (1.04‐1.38)	0.013

Stepwise logistic regressions were conducted to investigate the determinants of hypertensive TOD. Age and systolic blood pressure were forced into all models. Only variables staying in the final model are presented. ··· indicates nonsignificance; ba‐PWV indicates brachial‐ankle pulse‐wave velocity; cf‐PWV, carotid‐femoral pulse‐wave velocity; CIMT, carotid intima‐media thickness; CVD, cardiovascular diseases; OR, odds ratio; TOD, target organ damage. Definitions of hypertensive TOD were presented in Methods.

## Discussion

There were 2 major findings in the present study. First, both cf‐PWV and ba‐PWV exhibited similar associations with various cardiovascular risk factors in the elderly, including male sex, age, waist/hip circumference, fasting plasma glucose, and SBP. Second, hypertensive TOD, especially vascular and renal TOD, may be more closely associated with cf‐PWV than with ba‐PWV in the study cohort of community‐dwelling elderly.

For 2 decades, arterial stiffness was recognized as an important predictor of CVD and mortality. In theory, increased arterial stiffness is related to elasticity loss and reduced compliance in the arteries, which can further increase blood pressure.^27^ In turn, elevated blood pressure leads to vessel wall structure remodeling and vessel dysfunction to compensate for changes in wall stress, which can further exacerbate the arterial stiffness.^27^, ^28^, ^29^ Aging as an independent risk factor of hypertension and arterial stiffness also plays an important role in the vicious circle.^30^ In young individuals there is a stiffness gradient between the aorta and muscular arteries, which is reduced with increasing age through replacement of the degenerated elastic fibers by collagenous fibers in the vascular wall.^2^, ^31^ Additionally, intima‐media thickening, as a result of these modifications in vascular fibers, also influences arterial stiffening.^32^ Of note, it was known that aortic PWV increased nonlinearly and exponentially with aging, so it is possible that this process accelerated in the presence of hypertension and in the elderly.^5^, ^31^, ^33^, ^34^ In the present study we focused on subjects aged over 65 years to investigate the association of arterial stiffness estimated by cf‐PWV and ba‐PWV with cardiovascular risk factors and hypertensive TOD, aiming to identify the better indicator of arterial stiffening in the elderly.

Both cf‐PWV and ba‐PWV, as the most common indexes of arterial stiffness, have been widely used in clinical practice, with greater usage of cf‐PWV in the Western countries and greater usage of ba‐PWV in Asia.^4^, ^8^ For cf‐PWV, pressure transducers are placed on target arteries for the acquisition of carotid and femoral pressure waveforms and the calculation of the velocity. The resulting PWV is taken as representative of the PWV for the entire aorta. On the other hand, for ba‐PWV, there is no true arterial pathway linking the measurement sites (brachial to ankle). The resulting estimated PWV is taken as representative of the PWV for the entirety of the central and peripheral arterial system.^35^, ^36^ Both cf‐PWV and ba‐PWV were strongly linked with cardiovascular risk, and there was a positive association between cf‐PWV and ba‐PWV in previous publications.^1^, ^2^, ^35^, ^37^, ^38^, ^39^, ^40^ In accordance with these studies, we found that both cf‐PWV and ba‐PWV were significantly associated with male sex, aging, obesity, glucose profile, and high BP, and we also observed a significant and positive association between them in our present studies (data not shown). In addition, ba‐PWV was significantly associated with BMI. This finding may be attributable to the formula applied in the software built into the Omron ba‐PWV device and to the influence of adiposity on muscular arterial reflected wave in the elderly.

In literature,^2^, ^16^, ^41^, ^42^ cf‐PWV and ba‐PWV have been separately analyzed for their association with symptomatic TOD because hypertensive TOD is of great importance and is recognized as the intermediate outcome connecting cardiovascular risk factors and cardiovascular events and mortality. Because they are the 2 most common indicators of arterial stiffening in clinical practice, it makes sense to do a comparative analysis of cf‐PWV and ba‐PWV in their associations with TOD. Nevertheless, the magnitude of the association of TOD with cf‐PWV and ba‐PWV remains a subject of debate. In theory and based on the current guidelines for BP management, cf‐PWV was recognized as the golden standard of arterial stiffness as well as an important predictor of future cardiovascular risk.^5^ However, because ba‐PWV was more widely applied in Asia, some data suggested the opposite opinion. For instance, Yu et al^2^ indicated that, in a Chinese population, ba‐PWV correlated better with cardiac and vascular structure and function than cf‐PWV. On the contrary, we found that, in this community‐based elderly Chinese population, there was no significant difference between cf‐PWV and ba‐PWV in their association with cardiac TOD. However, cf‐PWV was more closely related to vascular and renal abnormalities than ba‐PWV, but with weak correlations and poor R^2^ values. The relatively small correlation coefficients and R^2^ values of the present study may be attributable to the complicated confounders and relatively weak association among the intermediate outcomes in the real‐world “healthy” elderly subjects. Nevertheless, it is also noteworthy that our findings were reliable because they were double checked by the multiple linear and logistic regression models and with many variables adjusted. As to the discrepancies existing between the cardiac and vascular/renal TOD in association with those 2 PWVs, the main reason responsible for them may be that cardiac structure and function indicated by LVMI and E/Ea were less affected by arterial stiffness as compared with vascular and renal abnormalities.

Our results were consistent with previous studies and current guidelines,^4^, ^5^ indicating the superiority of cf‐PWV over ba‐PWV in the association with vascular and renal abnormalities in the elderly. It is true that ba‐PWV can be more conveniently measured by an inexperienced or untrained practitioner, and accumulating data have indicated its significant prognostic value for cardiovascular events and mortality, such as the similar prospective study in a rural area of Shanghai.^16^ We indicated that, from the viewpoint of organ‐protection‐driven BP management, cf‐PWV, but not ba‐PWV, was recommended for the Chinese elderly, especially for those suffering from the vascular and renal abnormalities.

### Limitations

The findings of the present study need to be interpreted within the context of its limitations. First, as a cross‐sectional study, we focused only on the comparative analysis of cf‐PWV and ba‐PWV in association with cardiovascular risk factors and hypertensive TOD in the elderly but without any intervention or causality interpretation. With ongoing follow‐up studies, we will be able to provide more prospective data in the future. Second, we could not fully adjust for the influence of different medications on PWVs, such as various antihypertensive, antidiabetic, and antihyperlipidemic drugs, which may have differing effects on central and peripheral arterial stiffness.

### Perspectives

In light of the high burden of cardiovascular diseases on the aging society, an accurate assessment of arterial stiffness is of great importance and should be strongly recommended in the elderly. In the present study cf‐PWV was more closely associated with vascular and renal TOD in comparison with ba‐PWV in the elderly. However, the mechanisms remain incompletely understood. Therefore, further laboratory research is warranted to investigate the underlying mechanisms.

## Conclusions

Cf‐PWV seems to be superior to ba‐PWV in association with asymptomatic hypertensive TOD, especially vascular and renal TOD, in the community‐dwelling elderly Chinese population.

## Sources of Funding

This study was authorized and financially supported by the Shanghai Municipal Government (Grant ID 2013ZYJB0902; 15GWZK1002). Dr Yi Zhang was supported by the National Nature Science Foundation of China (Grant ID 81300239; 81670377).

## Disclosures

None.
